# Peptides Derived From the α-Core and γ-Core Regions of a Putative *Silybum marianum* Flower Defensin Show Antifungal Activity Against *Fusarium graminearum*

**DOI:** 10.3389/fmicb.2021.632008

**Published:** 2021-02-17

**Authors:** Agustina Fernández, María Laura Colombo, Lucrecia M. Curto, Gabriela E. Gómez, José M. Delfino, Fanny Guzmán, Laura Bakás, Ismael Malbrán, Sandra E. Vairo-Cavalli

**Affiliations:** ^1^CIPROVE-Centro Asociado CIC, Departamento de Ciencias Biológicas, Facultad de Ciencias Exactas, Universidad Nacional de La Plata, La Plata, Argentina; ^2^Consejo Nacional de Investigaciones Científicas y Técnicas (CONICET), Buenos Aires, Argentina; ^3^Instituto de Química y Fisicoquímica Biológicas (IQUIFIB), Universidad de Buenos Aires-CONICET, Buenos Aires, Argentina; ^4^Núcleo de Biotecnología Curauma (NBC), Pontificia Universidad Católica de Valparaíso, Valparaíso, Chile; ^5^Comisión de Investigaciones Científicas de la Provincia de Buenos Aires (CIC), Buenos Aires, Argentina; ^6^Centro de Investigaciones de Fitopatología (CIDEFI-UNLP-CIC), Facultad de Ciencias Agrarias y Forestales, Universidad Nacional de La Plata, La Plata, Argentina

**Keywords:** defensins, antimicrobial peptides, antifungal peptides, *Fusarium graminearum*, antifungal peptide design, fusarium head blight

## Abstract

*Fusarium graminearum* is the etiological agent of Fusarium head blight (FHB), a disease that produces a significant decrease in wheat crop yield and it is further aggravated by the presence of mycotoxins in the affected grains that may cause health problems to humans and animals. Plant defensins and defensin-like proteins are antimicrobial peptides (AMPs); they are small basic, cysteine-rich peptides (CRPs) ubiquitously expressed in the plant kingdom and mostly involved in host defence. They present a highly variable sequence but a conserved structure. The γ-core located in the C-terminal region of plant defensins has a conserved β-hairpin structure and is a well-known determinant of the antimicrobial activity among disulphide-containing AMPs. Another conserved motif of plant defensins is the α-core located in the N-terminal region, not conserved among the disulphide-containing AMPs, it has not been yet extensively studied. In this report, we have cloned the putative antimicrobial protein DefSm2, expressed in flowers of the wild plant *Silybum marianum*. The cDNA encodes a protein with two fused basic domains of an N-terminal defensin domain (DefSm2-D) and a C-terminal Arg-rich and Lys-rich domain. To further characterize the DefSm2-D domain, we built a 3D template-based model that will serve to support the design of novel antifungal peptides. We have designed four potential antifungal peptides: two from the DefSm2-D α-core region (SmAP_α1-21_ and SmAP_α10-21_) and two from the γ-core region (SmAP_γ27-44_ and SmAP_γ29-35_). We have chemically synthesized and purified the peptides and further characterized them by electrospray ionization mass spectrometry (ESI-MS) and Circular dichroism (CD) spectroscopy. SmAP_α1-21_, SmAP_α10-21_, and SmAP_γ27-44_ inhibited the growth of the phytopathogen *F. graminearum* at low micromolar concentrations. Conidia exposure to the fungicidal concentration of the peptides caused membrane permeabilization to the fluorescent probe propidium iodide (PI), suggesting that this is one of the main contributing factors in fungal cell killing. Furthermore, conidia treated for 0.5h showed cytoplasmic disorganization as observed by transmission electron microscopy (TEM). Remarkably, the peptides derived from the α-core induced morphological changes on the conidia cell wall, which is a promising target since its distinctive biochemical and structural organization is absent in plant and mammalian cells.

## Introduction

Wild plants and herbs provide a valuable source of antimicrobials because they exhibit a perfect adaptation to the environment, resulting in increased disease resistance relative to crop plants. Several highly active antimicrobial peptides (AMPs) have been isolated from different wild plant organs of the families Poaceae, Asteraceae, Ranunculaceae, and Caryophyllaceae (Astafieva et al., 2012). AMPs are important players in the immune response of plants against pathogens and pests; they comprise structurally diverse polypeptides that inhibit the growth of a wide range of microorganisms. Cysteine-rich peptides (CRPs) are AMPs particularly well represented in plants and they are divided into several families, including defensins. According to *in silico* studies from the analysis of complete plant genomes, most classes of cysteine-rich AMPs are much more abundant in the reproductive structures (Astafieva et al., 2012; Li et al., 2014). Although progress has recently been made recently in the identification of AMPs in seeds, those corresponding to flowers have been less studied (Astafieva et al., 2012). Concentrated in epidermal and stomatal cells, defensins are produced in areas that are likely to be the initial points of contact with pathogens (Parisi et al., 2018). Additionally, it was established that in the same species there are multiple defensin genes and this redundancy, necessary to protect the plant against selections of pathogens with higher tolerance to a particular type of defence molecule, is the result of the co-evolution of the immune systems of the plant and the pathogen (Vriens et al., 2014; Schmitt et al., 2016).

The main activity reported for plant defensins is antifungal, being active in micromolar concentrations (Hayes et al., 2013; Sagaram et al., 2013; Parisi et al., 2018). Besides antifungal activity, antibacterial, antiprotozoal and insecticidal action, inhibition of α-amylase, trypsin and protein synthesis as well as blockage of ion channels have been established for these molecules (Spelbrink et al., 2004; Lin et al., 2007; Vijayan et al., 2013; Nascimento et al., 2015; Parisi et al., 2018). Plant defensins do not only act against plant pathogens, some of these molecules are effective against human pathogens and tumour cells as well (Vriens et al., 2015; Bleackley et al., 2016).

Regarding their structure, all defensins have in common the presence in their sequences of several cysteines that form multiple disulphide bridges and share a common cysteine-stabilized 3-D fold (CSα/β) characterized by three antiparallel β-strands and one α-helix. In view of the 3-D conservation degree, any differences in defensin activity and specificity are likely to arise primarily from the considerable variation in the amino acid composition and the charge distribution of solvent-exposed loops. One of these loops is contained in the γ-core motif GXC(X_3–9_) C, a core pattern not limited to this defined AMP subclass but conserved across all classes of disulphide-stabilized AMPs. γ-core is a three-dimensional signature composed of two antiparallel β-sheets connected by a short turn region (Yount and Yeaman, 2004), which is considered the major determinant of antifungal activity of defensins (Sagaram et al., 2011; Muñoz et al., 2014). Defensins also contain a α-core motif with a consensus sequence GXC(X_3–5_)C not conserved in all disulphide-containing AMPs. This motif resides in the β1 strand-α-helix loop and contains part of the α-helix of each defensin; however, it lacks the hairpin structure of the γ-core motif (De Beer and Vivier, 2011; Sagaram et al., 2011).

More than 20% of global wheat (*Triticum aestivum* L.) production is lost annually due to the attack of pathogens and pests (Savary et al., 2019). Among the diseases affecting this crop in South America, Fusarium head blight (FHB), caused mainly by the fungal pathogen *Fusarium graminearum* Schwabe, is one of the most important (Savary et al., 2019). In Argentina, the frequency of occurrence of epidemics of FHB is currently increasing, with reported damages ranging from 20 to 70% (De Galich, 1997; De Ackermann and Kohli, 2013). Beyond the yield and quality losses caused by this disease, the commercial value of affected grains is further diminished by their contamination with mycotoxins, mainly the trichothecenes deoxynivalenol (DON), nivalenol (NIV), and their acetylated forms (McCormick, 2003). Ingestion of these compounds by humans and cattle frequently results in anorexia, depression of immune responses, nausea, vomiting, and/or necrosis of the gastrointestinal tract, bone marrow, and lymphoid tissues (Malbrán et al., 2018).

The challenge of this work was to achieve the identification of a new defensin from a wild species of the Buenos Aires flora (Asteraceae family) and to perform its *in silico* structural and functional characterization to support the design and synthesis of new peptides that could be attractive molecules for their potential agricultural or medical application. The designed peptides include those regions corresponding to the α-core or the γ-core motifs; their antifungal activity against the fungus *F. graminearum* was demonstrated and characterized. The use of the defensin sequence as a guide for peptide design could be a useful strategy for its potential application in the development of new antifungal compounds. It is well known that the γ-core motif contains major determinants of defensins antifungal activity and consequently it has already inspired the design of new antifungal compounds. The major contribution of this work was to demonstrate that the α-core region could also serve as a promising avenue for that purpose.

## Materials and Methods

### Biological Material

Flowers of *Silybum marianum* (L.) Gaertn. were collected during springtime from plants grown in La Plata, Buenos Aires (Argentina voucher specimen LPE 1162, Facultad de Ciencias Exactas, Universidad Nacional de La Plata, UNLP).

*Fusarium graminearum* SP1 was isolated from a grain sample obtained from San Pedro, Buenos Aires, Argentina. The strain was previously characterized as highly pathogenic and toxigenic both *in vitro* and *in vivo* (Malbrán et al., 2012, 2014).

### cDNA Cloning and Sequence Analysis

Closed flower buds of *S. marianum* were ground using a mortar and pestle chilled with liquid nitrogen. Total RNA was isolated using a commercial plant-specific kit (RNeasy® Plant Mini Kit, QIAGEN, Hilden, Germany) according to the manufacturer’s instructions. RNA was quantified by absorbance at 260nm, and its integrity was assessed in a 1% (w/v) agarose gel. Single-stranded cDNA was synthesized by retrotranscription of total RNA at 42°C for 60min using the M-MuLV RT enzyme (Thermo Fisher Scientific, Waltham, MA) in a 50μl reaction mixture containing reverse transcriptase buffer, dNTPs and the R_0_R_1_Oligo(dT)18 primer: 5'-CCGGAATTCACTGCAGGGTACCCAATACGACTCACTATAGGGCTTTTTTTTTTTTTTTT-3'. The cDNA synthesis control reaction consisted in amplifying the first strand with actin constitutive primers.

The resulting first strand of cDNA was subsequently used as a template for PCR amplification, using the degenerate forward primer 5'-AARAAYATHTGTGAAAAGCCAAGC-3' and the reverse primer (R_0_):5'-CCGGAATTCACTGCAG-3', designed from conserved N-terminal ends of Asteraceae. The PCR procedure consisted in an initial denaturation step at 94°C for 5min, followed by 35 cycles of denaturation at 94°C for 45s, annealing at 56°C for 47s, and extension at 72°C for 4min, with a final 15min extension at 72°C. The amplified fragments were cloned into a pGEM-Teasy vector (Promega, Madison, WI, United States) and used to transform *Escherichia coli* XL1-Blue competent cells. Plasmids from positive colonies were purified using a commercial kit (Wizard® Plus SV Minipreps DNA Purification System, Promega), and both strands were sequenced by automated DNA sequencing. The resulting sequences were subjected to multiple sequence alignment (MSA) using the Basic Local Alignment Search Tool (BLAST) algorithm from the National Centre for Biotechnology Information (NCBI, United States, https://blast.ncbi.nlm.nih.gov/Blast.cgi), and conserved domains were identified using NCBI’s conserved domain-Search service (Marchler-Bauer et al., 2011).

### Bioinformatic Analyses

Predicted defensin sequence (DefSm2-D) was used to perform a three-dimensional structural prediction by homology modelling. Fold assignment was performed using HHPred (Söding et al., 2005). Structural models were built with the program Modeller v 9.24 (Šali and Blundell, 1993) using ClustalW-derived alignments that were edited with GeneDoc software (version 2.7.000). NMR structure of Rs-AFP1 defensin from *Raphanus sativus* was used as template, Protein Data Bank^1^ code: 1AYJ. The quality of the model was assessed by using both energetic and structural criteria with PROSA II (Wiederstein and Sippl, 2007) and PROCHECK (Laskowski et al., 1993) software, respectively. Figures were drawn using PyMOL Molecular Graphics System (Version 1.2r3pre, Schrödinger, DeLano Scientific LLC, San Francisco, CA, United States). Functional prediction analysis was performed using the ProFunc server^2^ and the obtained structural model as input (Laskowski et al., 2005). The electrostatic Poisson-Boltzmann potential for the defensin structure was obtained through the APBS (Baker et al., 2001) molecular modelling software PyMOL with PARSE force field, optimized with the Python software package PDB2PQR (Dolinsky et al., 2004) and visualised in PyMOL.

### Peptide Design, Synthesis, and Purification

Identification of putative antifungal motifs in the sequence of DefSm2-D laid the foundation for the rational design of potential antimicrobial peptides. To this aim, MSA of defensins that have been reported to have essential regions for antimicrobial activity as well as the location of these regions in the three-dimensional model previously obtained from the cloned protein were considered. In turn, the peptides generated through the C-PAmP database (Niarchou et al., 2013) from sequences of available homologous defensins were also considered for the final design.

Peptides were synthesized using a Liberty Blue™ automated microwave peptide synthesizer (CEM Corp., Matthews, NC) following a standard 9-Fluorenyl methoxy carbonyl/tert-butyl (Fmoc/tBu) protocol. Fmoc-Rink Amide AM resin (Iris Biotech GmbH, Marktredwitz, Germany) 0.74mmol/g was used as solid support. Standard couplings of amino acids were carried out in N,N-dimethyl formamide (DMF) using N,N-diisopropylcarbodiimide (DIC)/OxymaPure® (Iris Biotech) activation and the corresponding amino acid. Fmoc removal was done with 20% v/v 4-methyl piperidine (4MP) in DMF. Peptides were cleaved with trifluoroacetic acid/triisopropylsilane/2,2-(ethylenedioxy)diethanethiol/ultrapure water TFA/TIS/DOT/H20: 95/2.5/2,5/2.5 under gentle agitation over a period of 3h at room temperature. After filtration, the crude peptides were precipitated by the addition of cold diethyl ether, centrifuged, washed five times with cold diethyl ether, and dried. Ten milligrams of each crude peptide were dissolved in water and loaded onto a Clean-Up® CEC18153 column (UCT, Bristol, PA), previously washed twice with methanol and twice with water. Elution was performed with successive mixtures of 10, 15, 20, 25, 30, and 60% (v/v) acetonitrile in water. Fractions collected were evaporated using a Savant SPD1010 SpeedVac Concentrator (Thermo Fisher Scientific). To determine the main fractions containing the expected peptide, reversed-phase (RP)-high performance liquid chromatography (HPLC) analysis was performed on a XBridge™ BEH C18 column (Waters Corporation, Milford, MA, United States) using a 0–70% acetonitrile gradient, with water containing 0.05% TFA as solvent A and acetonitrile containing 0.05% TFA as solvent B, at a flow rate of 1ml/min for 8min. The molecular mass of each peptide was determined by electrospray ionization mass spectrometry (ESI-MS) using a Shimadzu LCMS-2020 equipment (Shimadzu Corporation, Kyoto, Japan), in a 0–100% acetonitrile gradient for 20min. Peptides were stored as lyophilized dry powders at −80°C and dissolved just before use.

### Circular Dichroism Spectroscopy

Spectroscopic measurements were performed in the 180–250nm wavelength range (far UV) to determine the secondary structure of the DefSm2-derived peptides. Peptide samples were dissolved in water, and in a 25:75 (v/v) trifluoroethanol (TFE):H_2_O mixture at approximately 0.25mg/ml concentration, placed into a 1-mm path-length quartz cuvette at 25°C, and measured in a Jasco J-810 spectropolarimeter (Jasco, Inc., Easton, MD, United States) at a scan speed of 20nm/min and 1s time constant. Each spectrum in the plots results from the average of three successive scans, after applying a standard moving average window algorithm to smooth the trace. The solvent spectrum was measured similarly and subtracted from the corresponding spectra of peptides. Raw ellipticity data (mdeg) was converted to molar ellipticity, expressed in deg cm^2^ dmol^−1^ units.

### Fungal Growth Inhibition Assay

The peptides were tested for antifungal activity toward the filamentous fungi *F. graminearum* by performing hyphal growth inhibition assays according to the method of Bleackley et al. (2017) with slight modifications. Fungal isolates were grown under constant agitation on a carboxymethyl cellulose (CMC) sporulation medium (Cappellini and Peterson, 1965) at 170rpm and 25°C for 5–7days until spores were abundantly produced. Macroconidia were collected after centrifugation at 5000min^−1^ and 4°C for 15min, resuspended in half-strength potato dextrose broth (PD Broth) culture medium and adjusted to ≈5 × 10^4^ spores/ml using a hemocytometer. Aliquots (90μl) of the spore suspension were incubated for 48h at 25°C in a 96-well microplate with filter–sterilized peptide solutions (10μl) at different concentrations in water. Germination of spores was evaluated by measuring the optical density at 595nm using a microplate reader Infinite M200 Pro (Tecan, Männedorf, Switzerland) after 0, 19, 24, 43, and 48h of incubation. Each test was performed in triplicate. The minimum inhibitory concentration (MIC) was determined as the peptide concentration that completely inhibits fungal growth. Inhibition data were analysed by one-way ANOVA and the mean differences were evaluated at *p* < 0.05 using the Tukey test. Statistical analyses were performed using the InfoStat software (Di Rienzo et al., 2018).

For the most active peptides, a time-to-kill experiment was performed amending half strength PD Broth media with the peptides at their MIC. Half strength PD Broth (5ml) was inoculated with macroconidia from *F. graminearum* SP1 to a final concentration of 1 × 10^4^ conidia/ml. Peptides were added at their MIC and the amended PD Broth was cultured for different periods: 0.5, 1, 3, and 6h. A growth control was performed by incubating the conidia with water instead of peptide at 25°C in the dark for 48h. After each time period, 100μl of the 5ml culture were added to 900μl of sterilized water. This dilution was vortexed for 10s and 100μl were plated onto three different half strength potato dextrose agar (PD Agar) plates and incubated for 3days at 25°C in the dark before the colonies were counted. Each incubation time was replicated twice.

### Evaluation of Membrane Integrity

The effect of peptides at their MIC values on conidia membrane permeability of *F. graminearum* was assessed using the membrane impermeant fluorescent red dye propidium iodide (PI; Thermo Fisher Scientific). Spore suspensions (25μl; ≈10^7^ spores/ml in water) were amended with the peptide solutions prepared in water at their MIC and incubated for 30min at 25°C before visualization by fluorescence microscopy. An aliquot (5μl) of PI (0.1mM) was added to each suspension. After 30min of incubation at 25°C, the uptake of the fluorescence probe was evaluated at λ_ex_545nm and λ_em_ 580nm using an Olympus® BX-51 (Olympus Optical, Tokyo, Japan) fluorescence microscope. Photographs were taken with an Olympus® A330 (Olympus Optical) adapted digital camera. Spores that fluoresced red after incubation with PI were classified as damaged, whereas those unstained were classified as intact cells. Water and the commercial cationic surfactant cetyl trimethyl ammonium bromide (CTAB, 0.8mM; Cicarelli, Santa Fe, Argentina) were used as negative and positive controls, respectively. Each experiment, consisting of two replicates per treatment, was performed twice.

### TEM Imaging

Transmission electron microscopy (TEM) was used to study the effects of the most active peptides on the ultrastructure of spores. *F. graminearum* conidia (2 × 10^7^ml^−1^ in water) were exposed to the most active peptides at their MIC for 1h at 25°C and then prepared for electron microscopy imaging. The sample processing was carried out at room temperature and under gentle vacuum conditions (1atm). Conidia were fixed with 2.5% (v/v) glutaraldehyde in 0.1M sodium cacodylate buffer (pH 7.2) for 3h and postfixed for 2h with 1% (w/v) osmium tetroxide in 0.1M sodium cacodylate. Dehydration was carried out with aqueous ethanol solutions of increasing concentration (50 to 100% v/v). Finally, ethanol was replaced by acetone. The infiltration was performed with different proportions of acetone:spurr resin mixtures and the inclusion step was carried out in pure spurr resin. Ultrafine sections obtained by ultramicrotomy were contrasted with 1% (w/v) uranyl acetate and lead citrate. A negative control was performed with water. Images were taken with a JEM 1200 EXII (Jeol Ltd., Akishima, Tokyo, Japan) microscope located at the TEM Service, Facultad de Ciencias Veterinarias, UNLP.

## Results

### cDNA Cloning and *in silico* Analysis

**Figure 1:**
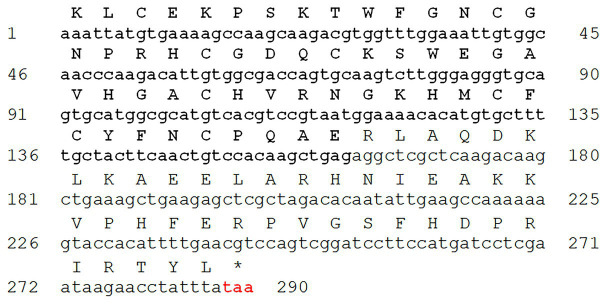
cDNA sequence and deduced amino acid sequence of DefSm2 (GenBank accession number: MK533801). Defensin-like domain (DefSm2-D) is shown in bold. Stop codon (TAA) is red colored.

To identify and further characterize *S. marianum* flower defensin, cDNA was synthesized using total RNA isolated from young flower buds by RT and the product of this reaction was used as template for PCR amplification. The 500bp amplified product was cloned, and several clones were sequenced. As a result, two different deduced amino acid sequences containing defensin domains were identified. These sequences exhibited high levels of similarity among themselves and with other plant defensins available in public databases. One of these cDNAs was selected for further characterization. Its nucleotide and deduced amino acid sequence are shown in Figure 1 (GenBank accession number: MK533801). This cDNA obtained encodes a protein of 95 amino acids containing an N-terminal defensin domain (DefSm2-D) with a cysteine arrangement that clearly matches that present in C8 defensins: C-X10-C-X5-C-X3-C-X[9-10]-C-X[6-8]-C-X-C-X3-C (Shafee et al., 2016) and a C-terminal Arg-rich and Lys-rich domain, of 54 and 41 amino acids, respectively. The deduced protein was designated as DefSm2. The BLAST Protein analysis of deduced amino acid sequence revealed that DefSm2-D showed 84% identity with DmAMP1 from seeds of *Dahlia merckii* (Accession Number P0C8Y4), 64% with AhAMP1 from seeds of *Aesculus hippocastanum* (Accession Number Q7M1F3) and 64% with Art v 1 from pollen of *Artemisia vulgaris* (Accession Number CBK62707). The predicted molecular weights of the complete protein, defensin, and its C-terminal domain were 10.95, 6.10, and 4.87kDa, respectively, and their corresponding isoelectric points were 9.01, 8.50, and 9.81.

**Figure 2:**
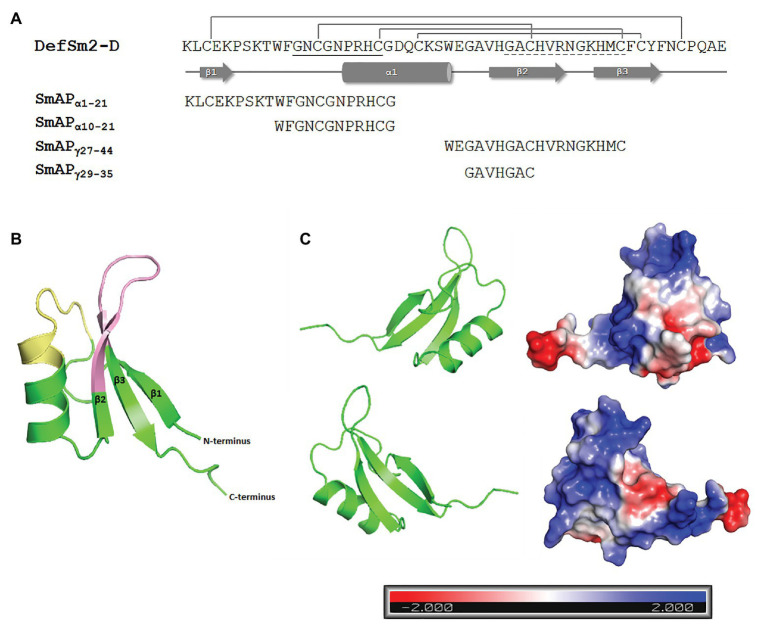
**(A)** Amino acid sequence and secondary structure prediction of DefSm2-D. The β strands are depicted with arrows and the α-helix is represented with a cylinder. Disulphide bonds between Cys3-50, Cys14-35, Cys20-44, and Cys24-46 are shown above. The α-core motif sequence is underlined with a continuous line, while the γ-core is underlined with a dashed line. The name and sequence of the synthesized peptides are shown below. **(B)** Homology-based model of DefSm2-D as a ribbon representation. The α-core motif is indicated in yellow and the γ-core is indicated in pink. 3-D model was built using Modeller and visualised with PyMOL as described in Materials and Methods. **(C)** Electrostatic surface potential of DefSm2-D visualised with PyMOL. Negative charge is indicated in red, neutral charge in white and positive charge in blue as shown in the scale below (isocontour value of ±2 kT/e). The protein is represented by two plots corresponding to a rotation of 180° around the vertical axis one from the other.

The amino acid sequence and the predicted 3D structure of DefSm2-D are shown in Figure 2A. As expected, deduced amino acid sequence analysis of DefSm2-D revealed the presence of the γ-core motif (GACHVRNGKHMC), presumably linked to antifungal activity, with four cationic residues. The analysis also revealed the presence of the α-core motif (GNCGNPRHC) with two cationic residues.

An acceptable overall quality score (Z-score: −5.18, according to PROSAII) was observed for the model, within the range characteristic for native proteins. The predicted 3D structure showed that most of the amino acid residues (98%) were positioned in energetically favoured or allowed regions, revealing a good stereochemistry for the model, according to the Ramachandran plot (Supplementary Figure 1). A characteristic defensin structure was observed: the typical cysteine-stabilized alpha-beta (CSαβ) fold (Figure 2B). The structural model contains three antiparallel β-strands and one α-helix connected by four disulphide bridges (Cys3-50, Cys14-35, Cys20-44, and Cys24-46). The γ-core motif resides in a hairpin loop between β2 and β3 strands, while the α-core region comprises the Pro turn at the N-terminus of the α-helix and the proximal part of the loop connecting it to the β1-strand (Figure 2B). Both regions show a predominantly positive electrostatic surface potential (Figure 2C).

Based on the analysis carried out with the ProFunc server, the most frequent Gene Ontology (GO) terms describing the putative function of the DefSm2D domain (*cellular component*, *biological process*, and *molecular function*) could be identified. Some of the most significant results for each GO term were extracellular component, defence, stress, and biotic stimulus response, the killing of cells from another organism and antifungal response.

### Peptide Design, Synthesis, and Characterization

The analysis of the potential antimicrobial peptide list generated with C-PAmP, using the sequences of the defensins with higher identity with DefSm2-D as input, showed that the predicted peptides were in the α-core or γ-core regions of the protein. The score of the peptides located around both regions, as well as their position in the 3D structure of DefSm2-D, were considered for peptide design. Four peptides were synthesized through the F-moc strategy: two from DefSm2-D α-core sequence (called SmAP_α1-21_ and SmAP_α10-21_), one from the γ-core (called SmAP_γ27-44_), and one smaller peptide contained in the latter (called SmAP_γ29-35_). Table 1 summarizes the main characteristics of the designed peptides. The peptide SmAP_α1-21_ includes four additional charged amino acid residues: three cationic (Lys) and one anionic (Glu) as compared to SmAP_α10-21_.

**Table 1:** Main properties of DefSm2-D derived peptides.

Peptide name	Sequence	Molecular weight (Da)	pI^1^	Net charge^2^	MIC (μM)^3^
SmAP_α1-21_	KLCEKPSKTWFGNCGNPRHCG	2361.7	9.0	4	32
SmAP_α10-21_	WFGNCGNPRHCG	1346.5	8.4	1.9	70
SmAP_γ29-35_	GAVHGAC	613.9	6.7	0.9	-
SmAP_γ27-44_	WEGAVHGACHVRNGKHMC	1991.3	8.1	3.8	20

1 Theoretical pI was calculated using the ExPASy tool Compute pI/Mw.

2 Net charge was calculated at pH 5.5 (pH of half strength PDB in which the antifungal test was performed).

3 Minimum inhibitory concentration (MIC) is considered the minimal peptide concentration that completely inhibits *F. graminearum* growth.

Consistent with the lack of structure, the overall shape of CD spectra reveals that peptides adopt a predominant random coil conformation in water (Supplementary Figure 2). Trifluoroethanol is widely recognized as a useful co-solvent to assess the secondary structure propensity of peptides and proteins. However, its general mode of action is to promote (α-helix and β-hairpin) secondary structure formation in polypeptides. After defying the molecules with this co-solvent, their far UV-CD spectra were measured to assess their structural behaviour. The SmAP_α1-21_ and SmAP_α10-21_ peptides, mapping to the α-core sequence, show minimal enhancement of negative ellipticity bands located in the range of 215–230nm, pointing to a marginal gain of structure effect upon the TFE challenge. Nonetheless, for all four peptides the overall pattern of the far UV-CD bands, both in position and intensity, was maintained even at the high TFE concentration assayed (25% v/v). These results are consistent with intrinsic fluorescence emission spectra, which reveal that in all tryptophan-containing peptides this residue is mainly exposed to the aqueous solvent (results not shown).

### Antifungal Activity Assays

**Figure 3:**
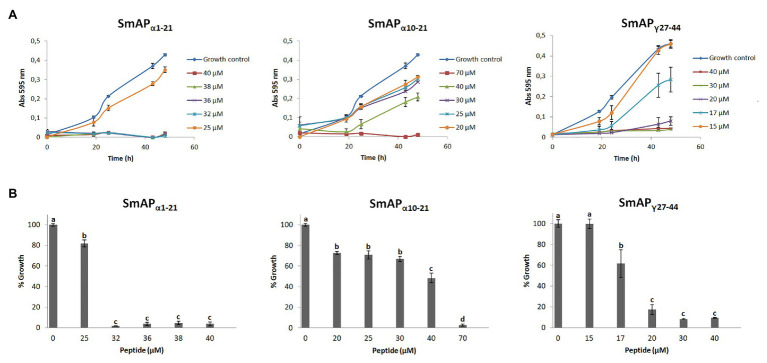
**(A)** Growth curves of *Fusarium graminearum* in the presence of different concentrations of DefSm2-D derived peptides. Error bars represent the SD of technical triplicates. Abs 595nm is the absorbance at 595nm. **(B)** Percentage of growth of *F. graminearum* at the final time point in panel A (48h). Bars represent the mean ± the SD of the percentage of growth as compared to the 100% from the control growth, defined as the fungus growth in the absence of peptide. Treatments with the same letter do not differ significantly (*p* > 0.05).

The changes in the optical density over time in the presence of increasing concentrations of each peptide are shown in Figure 3A. Three peptides produced significant fungal inhibition when compared to the growth control (Figure 3B). Peptide SmAP_γ29-35_ was tested at concentrations up to 100μM but did not show any antifungal activity (data not shown). For the remaining peptides, at concentrations under the MIC, exposure to increasing peptide concentrations does not affect the duration of the lag phase of the turbidimetric growth curves. As expected, comparison between both α-core derived peptides revealed that the longer peptide is more effective than the shorter one.

In the time to kill experiment, SmAP_α1-21_ and SmAP_γ27-44_ were lethal for conidia (Supplementary Figure 3). These peptides were able to exert their activity within 0.5h of incubation at the assay conditions.

**Figure 4:**
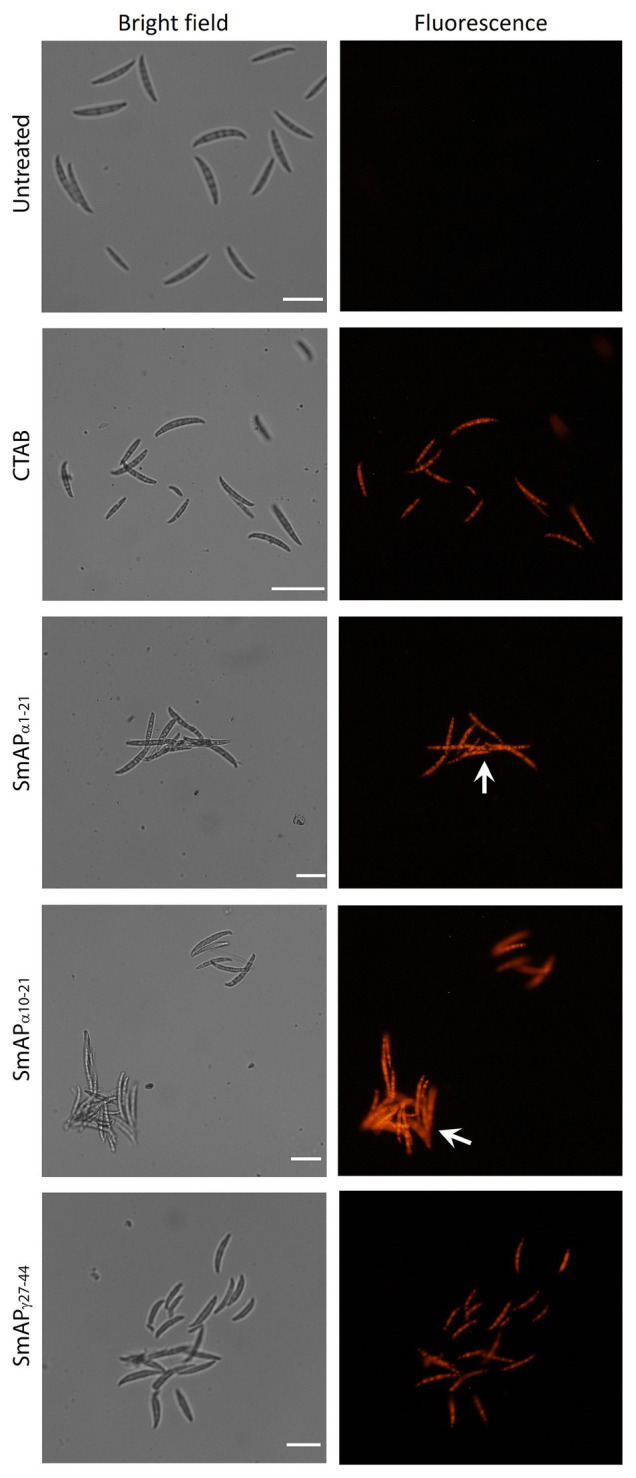
Bright field (left) and fluorescence (right) images of *F. graminearum* conidia incubated with peptides at their MIC for 1h. CTAB is the cationic surfactant cetyl trimethyl ammonium bromide, used as positive control. Peptides from the α-core region, SmAP_α1-21_ and SmAP_α10-21_, produced the aggregation of conidia in clusters (white arrows). Scale Bar = 20μM.

In addition, the uptake of PI was visible in fungal cells treated with SmAP_α1-21_, SmAP_α10-21_, and SmAP_γ27-44_ (Figure 4). Interestingly, in the presence of the α-core derived peptides macroconidia aggregate into network-like clusters (Figure 4, white arrows), which are more prominent for cells treated with SmAP_α1-21_ than for those treated with SmAP_α10-21_. Although SmAP_γ27-44_ and the surfactant used as control also induce permeabilization of the *F. graminearum* spore membrane, clusters are not formed under any of these treatments.

**Figure 5:**
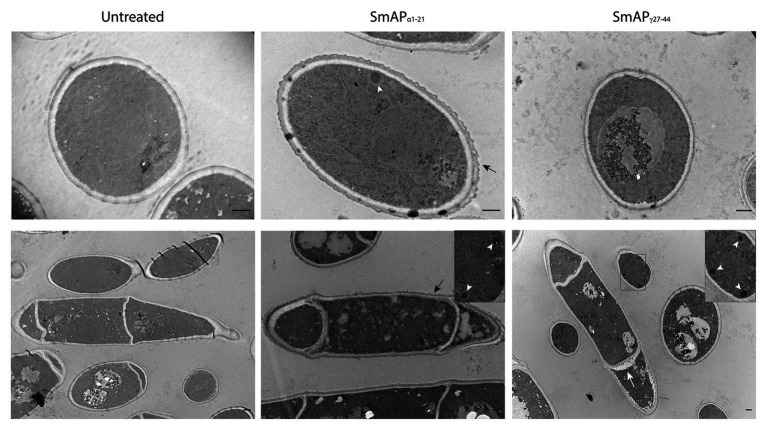
Transmission electron microscopy (TEM) images of *F. graminearum* conidia incubated with peptides SmAP_α1-21_ and SmAP_γ27-44_ at their MIC for 30min. A scalloped appearance was observed around the cells treated with SmAP_α1-21_ (black arrows). With SmAP_γ27-44_, the cortical cytoplasm is separating from the cell wall (white arrow). In both cases, an increased electron density and the presence of electron-dense peroxisomes (zoom-in detail, white arrowheads) compared with the untreated cells were observed. Scale bar = 0.5μm.

Transmission electron microscopy images of conidia exposed to SmAP_α1-21_ reveal a scalloped appearance around the cells (Figure 5, black arrows). In addition, the cells present a granular and disorganized cytoplasm, suggesting that this peptide exerts a remarkable effect on the cell wall outermost layer. SmAP_γ27-44_ does not produce the same effect, but some signs of cell deterioration could be seen, including segregation of the cytoplasm from the cell periphery (Figure 5, white arrow). In both cases, an increased electron density and the presence of a large number of electron-dense peroxisomes (Figure 5, white arrowheads) were observed with respect to the untreated cells.

## Discussion

The massive use of antifungal agents in the agriculture sector has led to the emergence of fungicide-resistant strains, restricting the number of commonly used compounds of this kind (Brauer et al., 2019) and probably contributing to developing resistance against antifungal medicines (Jampilek, 2016; Perfect, 2016). For FHB in particular, several factors further hinder the effectiveness of fungicide treatments, including lack of complete control by the available molecules, uneven flowering of wheat, and poor retention of fungicides on the spikes (Wegulo et al., 2015). In this context, there is an urgent need to search for new safe and effective antifungals. Wild plants and weeds naturally exhibit an enhanced pathogen resistance due to their perfect environment adaptation, constituting a valuable albeit unexplored source of natural AMPs (Slavokhotova et al., 2011) such as defensins. The wide range of pathogens that AMPs are active against and the varying levels of effectiveness that they exhibit could be attributed to the fact that these peptides have taken advantage of biochemical divergence and evolution of the cell wall and cell membrane composition, acting preferentially on pathogens whereas being harmless to the host (Teixeira et al., 2012). In view of its future applicability, a deep insight into the structure and function of natural AMPs is essential for future protein engineering and rational design of improved AMPs in view of its future applicability.

In this study, we report the identification of DefSm2, a putative antifungal protein with a defensin domain naturally expressed in flowers of the wild thistle *S. marianum*. Flowers are nutrient-rich structures that contain an abundance of protection molecules that serve as a front-line defence in peripheral cell layers (Silverstein et al., 2007). The *in silico* analysis of the cDNA derived-amino acid sequence of DefSm2 revealed the presence of two fused basic domains; the N-terminal domain is a C8 defensin, while the C-terminal domain is rich in Arg and Lys. Of still unknown function, CRP fusions frequently occur in plant genomes and transcriptomes with other CRP, glycine-, or proline-rich protein domains. De Paula et al. (2011) described for Sd5, a defensin from *Saccharum officinarum*, the presence of an unstructured C-terminal region that has not been identified in any plant defensin structure reported in the Protein Data Bank with a predominance of hydrophobic amino acids as well as charged residues (Arg, Glu, and Lys) that participate in defensin-membrane interaction. A Lys-rich C-terminal domain was found in Ha-DEF1, a defensin from sunflower (Asteraceae) leaf and root (Letousey et al., 2007). By searching for homologous sequences, we have found predicted defensins in other species of the Asteraceae family fused to cationic C-terminal domains but just like for Ha-DEF1, those domains are shorter and have fewer cationic amino acids than DefSm2 C-terminal domain. Moreover, even though DefSm2-D shares high identity with predicted defensin domains from fused proteins of *Cynara cardunculus* var. *scolymus* (accession numbers XP_024967363 and XP_024968136), the DefSm2 C-terminal domain shares lower or no sequence identity with their predicted basic C-terminal domains. This intriguing and unique domain should have a precise function in *S. marianum*, a matter that still deserves proper consideration.

As CRP AMPs, plant defensins are held together by four disulphide bonds. The spacing and connectivity of the highly conserved cysteine residues define the plant defensin family (Shafee et al., 2016). According to our model, DefSm2-D disulphide connectivity is 1:8, 2:5, 3:6, and 4:7, which is common to C8 defensins. Disulphide bonds confer the molecule high resistance to proteases and to extreme values of pH and temperature, which is a valuable trait in the front line of the host-pathogen battle (De Coninck et al., 2013; Parisi et al., 2018). Furthermore, this disulphide-bonding network stabilizing the correct fold also seems to be central for many of defensin activities (Powers et al., 2005; Dhople et al., 2006). Most likely, this arrangement would facilitate the surface exposure of specific amino acid residues in the intervening loops, contributing to the establishment of interactions key for antimicrobial activity.

Notwithstanding their conserved tertiary structure, plant defensins share very low identity at the amino acid level. This sequence variability contributes to the different biological functions that have been attributed to these proteins, where a change of a single amino acid can alter the spectrum of activity exhibited by closely related defensins and can also account for their diverse antifungal modes of action (Thomma et al., 2003; Thevissen et al., 2007; Aerts et al., 2008). Amino acid residues at typically conserved positions are two glycine residues, an aromatic amino acid, and a glutamic acid (Ghag et al., 2016); the position numbers relative to DefSm2-D are Gly12 and Gly33, Trp10, and Glu28. It was proposed that these residues also contribute to the defensin structure stabilization and that the highly conserved Gly12 is present in all plant defensins, contributing to the plasticity of the loop, which is probably essential for the recognition process (De Medeiros et al., 2010). As expected, the deduced amino acid sequence analysis of DefSm2-D revealed the presence of the γ-core motif. Interestingly, we have found that this is identical to the DmAMP1 γ-core motif. DmAMP1 is active against *Neurospora crassa*, *Fusarium solani*, *Fusarium culmorum* (Osborn et al., 1995), and *Saccharomyces cerevisiae* among other fungi. On the other hand, the α-core motif is also present in DefSm2-D, but it is not conserved in all disulphide-containing AMPs and differs from the β-hairpin structure of the γ-core motif. Although the overall similarity among defensin sequences is small, the loops where the α and γ-core are located constitute interacting regions with pathogen surface structures that can reach high identity scores when compared to defensins that share the same membrane target (De Medeiros et al., 2010). The Arg38 residue contained in the γ-core motif, although not conserved in all plant defensins, was reported to be crucial for the antifungal activity of MsDef1 against *F. graminearum* (Sagaram et al., 2011). Moreover, Arg38 is not only an important residue for antifungal activity in plant defensins but also for membrane interaction of human defensins with membranes of both Gram-positive and Gram-negative bacteria (Lee et al., 2016). Remarkably, this residue is present in DefSm2-D, and the derivative peptide SmAP_γ27-44_ containing this Arg residue was the most active peptide against the aforementioned pathogen. Although positive charge prevalence is observed on the surface of DefSm2-D, according to our model, areas with a negative charge density are also observed, pointing to the small number of acidic residues also contributing to the polar face.

Many efforts have been made to identify minimal active motifs in defensins that would allow to creating new antimicrobial agents (De Medeiros et al., 2010). In fact, there are studies reporting a higher activity from peptides derived from a full-length sequence than the source itself (Schaaper et al., 2001). Recently, Sathoff et al. (2019) showed that chemically synthesized peptides containing the γ-core motif of defensins may mimic the relative biological activity of the full-length defensin. On the contrary, Garrigues et al. (2017) found that PAF109, a peptide designed from the γ-core motif of the antifungal protein AfpB of fungal origin, does not show antifungal activity against several fungi evaluated. Muñoz et al. (2014) found that the synthetic peptides derived from the γ-core motif of MtDef4 and MsDef1 (from *Medicago truncatula* and *Medicago sativa*, respectively) possess specific antifungal properties that differ from those of the parental defensin. However, the fragmentation in many antimicrobial proteins and peptides can also generate activities that would not represent that of the parental molecule. In this work, we have designed four synthetic peptides from the α- and γ-core motifs of DefSm2-D, demonstrating the ability in three of them to inhibit the growth of the phytopathogen *F. graminearum*. Arg, Lys, as well as, His residues, are distributed along the regions selected for the design of SmAP_α10-21_, SmAP_α1-21_, and SmAP_γ27-44_. Compared to SmAP_α10-21_, the peptide SmAP_α1-21_ includes three extra cationic amino acids (Lys) and one anionic amino acid (Glu). A Trp residue is also present in the three active peptides synthesized, which is considered to play a pivotal function in the partition of AMPs in the membrane, anchoring the peptide at the membrane interface (Teixeira et al., 2012). In SmAP_α1-21_ and SmAP_α10-21_, Phe is adjacent to Gly, the first residues of the α-core motif. In peptide SmAP_γ27-44_, Phe is not present and the hydrophobicity, besides Trp, is ensured by the presence of Val and Met. The cationic amino acids would establish electrostatic interactions with negatively charged pathogen membranes or cell wall, while hydrophobic amino acids would contribute to the interactions at the lipid membrane interface in the target pathogen cell. Regardless of the specifics of the AMP mechanism of action, the first step appears to require site-specific binding targets on the pathogen cell wall and/or membrane (El-Mounadi et al., 2016; Cools et al., 2017). Circular dichroism evidence indicates that all peptides assayed remain unstructured in solution, with scant or no propensity to adopt a definite secondary structure (Supplementary Figure 2). Quite reasonably, the maintenance of plasticity along these stretches would enhance molecular recognition with target sites, in a picture fully consistent with the location of these dynamic and exposed motifs in the parent protein.

Much less studied is the role of the α-core motif in antifungal activity and, to our knowledge, this is the first instance where peptides derived from this motif and its adjacent regions were shown to prevent fungal growth at micromolar concentrations. The α-core motifs from MsDef1 and MtDef4 defensins were also chemically synthesized and tested for antifungal activity. Both GPCFSGC and GPCASDHNC peptides were totally inactive at all concentrations assayed, indicating that the α-core motifs of MsDef1 and MtDef4 *per se* do not exhibit antifungal activity. Peptides derived from the α-core region from *Brassica hybrid* cv. Pule defensin exhibited activity against *Colletotrichum gloeosporioides* albeit at millimolar concentrations (Kaewklom et al., 2016). Probably, the α- and γ-core motifs require the presence of adjacent residues to display antifungal activity. Muñoz et al. (2014) found that the γ-core from MsDef1, a defensin active against *N. crassa*, does not show antifungal activity by itself, but a peptide spanning the γ-core six residues (including Trp, Arg, and Lys) of the defensin shows high inhibitory activity. In contrast, the γ-core from MtDef4, which presents three Arg residues, is almost as effective as the full defensin molecule against the above-mentioned fungus and the addition of extra amino acids towards the C-terminal end does not affect its biological activity. We decided to span the α- and γ-core from DefSm2-D towards the N-terminal region in both cases, due to the presence of residues Lys and Trp that could potentially enhance antimicrobial activity. In fact, an increase in activity was verified for the peptide SmAP_α1-21_ compared to SmAP_α10-21_. SmAP_α1-21_ contains the SmAP_α10-21_ sequence but in addition it presents a higher net cationic charge given by the presence of three extra Lys residues. Similarly, two peptides of 10 amino acids from the N-terminal region of a rice defensin (OsAFP1) were shown to be active against *Candida albicans* at micromolar concentrations. These peptides were designed among others as overlapping peptides from different regions that collectively covered the entire OsAFP1 sequence. Here again, the active N-terminal peptides included both hydrophobic and cationic residues (Ochiai et al., 2018).

Using the PI uptake assay, we showed that peptides SmAP_α1-21_, SmAP_α10-21_, and SmAP_γ27-44_ permeabilized the plasma membrane of *F. graminearum* conidia. This dye penetrates into cells with damaged membranes and binds to nucleic acids, being therefore indicative of the loss of cell viability. All the conidia tested internalize the PI within 0.5h of incubation when treated with peptides at their MICs, suggesting that membrane permeabilization is either a contributing factor for the antifungal activity of these peptides or a consequence of their action. Furthermore, short incubation periods in the presence of these peptides are enough to prevent conidia germination. In contrast with many reports of membrane permeabilization of fungi hyphae caused by defensin or derived peptides, our assays were performed on conidia that are resistant fungal structures endowed with thick cell walls, emphasizing the promising antimicrobial activity of the *S. marianum* DefSm2-D derived peptides. The presence of Phe and/or Trp as “hydrophobic anchors” and cationic residues could play a key role in membrane permeabilization. In this regard, Sagaram et al. (2011) clearly demonstrated the importance of the Phe residue in hexapeptide RGFRRR, which causes hypha permeabilization in *F. graminearum*, whereas the variant RGARRR is totally ineffective. Similarly, we propose that these aromatic hydrophobic residues bear an important role in the antifungal activity of SmAP_α1-21_, SmAP_α10-21_, and SmAP_γ27-44_, as attested by the lack of activity shown for SmAP_γ29-35_.

The effect of cell aggregation was recently observed by Velivelli et al. (2018) on the bacteria *Xanthomonas campestris* treated with MtDef5B, one of the two defensin domain from *M. truncatula*. These authors also found that the simultaneous replacement of His-Arg by Ala-Ala in the two γ-core motifs of MtDef5 (H36 and R37 in domain A and H93 and R94 in domain B) leads to a loss of bacterial cell killing effect, prevents the build-up of aggregates and induces the formation of bacteria chain-like structures. In SmAP_γ27-44_, although an Arg residue is present equivalent to the Arg 37 position in MtDef5 there is no adjacent His and no visible aggregation is observed in the PI uptake assay. Conversely, the peptides derived from the DefSm2-D α-core that present a His residue adjacent to Arg (R18 and H19 relative to DefSm2-D numbering) induce conidia aggregation.

The aggregation effect of the α-core peptides on conidia could be further explained by the modifications on the conidia ultrastructure observed by TEM. Fungal cell walls are complex and dynamic structures, essential for cell viability, morphogenesis and pathogenesis, and are associated to many enzymes and metabolic pathways. They consist of an electron-lucent innermost layer, comprising a relatively conserved structural skeleton of chitin and β-glucan electron-dense and heterogeneous outer layers (Gow et al., 2003). TEM results indicate that this outermost protective layer would be the one most likely affected by the action of SmAP_α1-21_. The fungal cell wall constitutes a promising target for the development of antifungal compounds due to its unique biochemical and structural organization, which is absent in plant and mammalian cells. The effect of the peptides on several potential molecules including chemical components of the outermost layer, enzymes involved in the remodelling of the outer cell wall zone or messengers that trigger the observed fungal response could explain their antifungal effects.

The outer cell wall of *F. graminearum* conidia is formed by layers of α-(1,3) glucan with hydrophobic proteins called hydrophobins, which probably form a cover of rodlets that protect the spores (Quarantin et al., 2019). In *Aspergillusfumigatus*, a similar “rodlet layer” surface prevents immune recognition by both innate and adaptative immune defence systems in mammals (Aimanianda et al., 2009). In plant fungal infection, hydrophobins are required to penetrate the water-air interface and to attach to hydrophobic surfaces, such as the spike tissue. Furthermore, the simultaneous presence of different hydrophobins on the *F. graminearum* conidia surface could form a protecting shield against toxic compounds, turning this structure into a potential target for antifungal compounds. Regarding the enzymes responsible for remodelling and maintaining the cell wall, fungal cell death could be the result of the inhibition of the cell wall polysaccharide synthases (Latgé, 2007).

For SmAP_γ27-44_, no apparent morphological changes were seen on the cell wall, but the observed shrunk cytoplasm detached from the cell wall might be indicative of early stage cell deterioration. A similar effect was observed by Sagaram et al. (2013) when treating *F. graminearum* hyphal cells with the defensin MtDef4. Moreover, for both SmAP_α1-21_ and SmAP_γ27-44_ peptides, a granulated electron-dense cytoplasm was observed, similarly to that observed with MtDef4 and Nad1 on *F. graminearum* and on *F. oxysporum* hyphal cells, respectively (van der Weerden et al., 2008; Sagaram et al., 2013).

Fungal pathogens present a remarkable genetic flexibility that contributes to the rapid evolution and adaptation to the host and the environment. To avoid the emergence of resistant strains, the development of new antifungal agents is needed. In this study, we report the antifungal activity of three synthetic peptides derived from the α- and γ-core region of DefSm2-D from the wild plant *S. marianum* against *F. graminearum* conidia. According to our results, for SmAP_α1-21_ derived from the α-core, a fungal cell wall component could presumably act as the first peptide binding target site. Although this is not the most active peptide, we believe it is an interesting starting point for further development of new antifungal agents that might act on the fungal cell wall, a key structure in the pathogen resistance and in the evasion of host response. Furthermore, the differential cell wall composition of the pathogen with respect to the host would account for a selective action of the peptides on the fungus and not on the host cell. SmAP_γ27-44_ instead, might induce fungal cell death through a different mechanism, probably independent of the cell wall target. Regardless of their precise mechanism of action, amino acid composition of the active peptides suggests that positively charged combined with aromatic amino acid residues are signature entities obligatory to attain antimicrobial activity. In this regard, our peptides would constitute promising antifungal agents since they are active *in vitro* against a resistant fungal structure such as the conidia. Further research is required in order to gain insight into the mechanism of action of these peptides.

## Data Availability Statement

The datasets presented in this study can be found in online repositories. The names of the repository and accession number can be found at: GenBank from the National Center for Biotechnology Information^3^ with the accession number MK533801 for DefSm2.

## Author Contributions

AF, MC, and SV-C: defensin cloning. AF, SV-C, and JD: bioinformatic analyses. AF, SV-C, and LB: peptide design and paper writing. FG: peptide synthesis and purification. LC, GG, and JD: circular dichroism spectroscopy. AF and IM: antifungal assays. AF and SV-C: TEM imaging. All authors contributed to the article and approved the submitted version.

### Conflict of Interest

The authors declare that the research was conducted in the absence of any commercial or financial relationships that could be construed as a potential conflict of interest.

## Footnotes

^1^www.rcsb.org

^2^http://www.ebi.ac.uk/thornton-srv/databases/profunc/

^3^https://www.ncbi.nlm.nih.gov/genbank/

## References

[ref1] Aerts A. M. François I. E. J. A. Cammue B. P. A. Thevissen K. (2008). The mode of antifungal action of plant, insect and human defensins. Cell. Mol. Life Sci. 65, 2069–2079. 10.1007/s00018-008-8035-0, PMID: 18360739 PMC11131863

[ref2] Aimanianda V. Bayry J. Bozza S. Kniemeyer O. Perruccio K. Elluru S. R.. (2009). Surface hydrophobin prevents immune recognition of airborne fungal spores. Nature 460, 1117–1121. 10.1038/nature08264, PMID: 19713928

[ref3] Astafieva A. A. Rogozhin E. A. Odintsova T. I. Khadeeva N. V. Grishin E. V. Egorov T. A. (2012). Discovery of novel antimicrobial peptides with unusual cysteine motifs in dandelion *Taraxacum officinale* wigg. flowers. Peptides 36, 266–271. 10.1016/j.peptides.2012.05.009, PMID: 22640720

[ref4] Baker N. A. Sept D. Simpson J. Holst M. J. McCammon J. A. (2001). Electrostatics of nanosystems: application to microtubules and the ribosome. Proc. Natl. Acad. Sci. U. S. A. 98, 10037–10041. 10.1073/pnas.181342398, PMID: 11517324 PMC56910

[ref5] De Beer A. Vivier M. A. (2011). Four plant defensins from an indigenous south african brassicaceae species display divergent activities against two test pathogens despite high sequence similarity in the encoding genes. BMC Res. Notes 4:459. 10.1186/1756-0500-4-459, PMID: 22032337 PMC3213222

[ref6] Bleackley M. Dawson C. S. McKenna J. A. Quimbar P. Hayes B. M. E. van der Weerden N. L.. (2017). Synergistic activity between two antifungal proteins, the plant defensin NaD1 and the bovine pancreatic trypsin inhibitor. mSphere 2, e00390–e00417. 10.1128/mSphere.00390-17 PMC5646242 29062897

[ref7] Bleackley M. R. Payne J. A. E. Hayes B. M. E. Durek T. Craik D. J. Shafee T. M. A.. (2016). *Nicotiana alata* defensin chimeras reveal differences in the mechanism of fungal and tumor cell killing and an enhanced antifungal variant. Antimicrob. Agents Chemother. 60, 6302–6312. 10.1128/AAC.01479-16, PMID: 27503651 PMC5038239

[ref8] Brauer V. S. Rezende C. P. Pessoni A. M. De Paula R. G. Rangappa K. S. Nayaka S. C.. (2019). Antifungal agents in agriculture: friends and foes of public health. Biomolecules 9:521. 10.3390/biom9100521, PMID: 31547546 PMC6843326

[ref9] Cappellini R. A. Peterson J. L. (1965). Macroconidium formation in submerged cultures by a non-sporulating strain of gibberella zeae. Mycologia 57, 962–966. 10.2307/3756895

[ref10] Cools T. L. Struyfs C. Cammue B. P. Thevissen K. (2017). Antifungal plant defensins: increased insight in their mode of action as a basis for their use to combat fungal infections. Future Microbiol. 12, 441–454. 10.2217/fmb-2016-0181, PMID: 28339295

[ref11] De Ackermann M. D. Kohli M. M. (2013). “Chemical control of fusarium head blight of wheat” in Fusarium head blight in latin America. eds. Teresa M A. M. Noemí C. S. (Dordrecht, Netherlands: Springer Netherlands), 175–189.

[ref12] De Coninck B. Cammue B. P. A. Thevissen K. (2013). Modes of antifungal action and in planta functions of plant defensins and defensin-like peptides. Fungal Biol. Rev. 26, 109–120. 10.1016/j.fbr.2012.10.002

[ref13] De Galich M. T. V. (1997). “Fusarium head blight in Argentina” in Fusarium head scab: Global status and future prospects. eds. Dubin H. J. Gilchrist L. Reeves J. McNab A. (México: CIMMYT México D.F.), 19–28.

[ref14] De Medeiros L. N. Angeli R. Sarzedas C. G. Barreto-Bergter E. Valente A. P. Kurtenbach E.. (2010). Backbone dynamics of the antifungal Psd1 pea defensin and its correlation with membrane interaction by NMR spectroscopy. Biochim. Biophys. Acta 1798, 105–113. 10.1016/j.bbamem.2009.07.013, PMID: 19632194

[ref15] De Paula V. S. Razzera G. Barreto-Bergter E. Almeida F. C. L. Valente A. P. (2011). Portrayal of complex dynamic properties of sugarcane defensin 5 by NMR: multiple motions associated with membrane interaction. Structure 19, 26–36. 10.1016/j.str.2010.11.011, PMID: 21220113

[ref16] Dhople V. Krukemeyer A. Ramamoorthy A. (2006). The human beta-defensin-3, an antibacterial peptide with multiple biological functions. Biochim. Biophys. Acta 1758, 1499–1512. 10.1016/j.bbamem.2006.07.007, PMID: 16978580

[ref17] Di Rienzo J. A. Casanoves F. Balzarini M. Gonzalez L. A. Tablada M. Robledo C. W. (2018). “InfoStat.” Córdoba, Argentina: Grupo InfoStat, FCA, Universidad Nacional de Córdoba. Available at: http://www.infostat.com.ar (Accessed May 15, 2020).

[ref18] Dolinsky T. J. Nielsen J. E. McCammon J. A. Baker N. A. (2004). PDB2PQR: an automated pipeline for the setup of poisson-boltzmann electrostatics calculations. Nucleic Acids Res. 32, W665–W667. 10.1093/nar/gkh381, PMID: 15215472 PMC441519

[ref19] El-Mounadi K. Islam K. T. Hernández-Ortiz P. Read N. D. Shah D. M. (2016). Antifungal mechanisms of a plant defensin MtDef4 are not conserved between the ascomycete fungi *Neurospora crassa* and *Fusarium graminearum*. Mol. Microbiol. 100, 542–559. 10.1111/mmi.13333, PMID: 26801962

[ref20] Garrigues S. Gandía M. Borics A. Marx F. Manzanares P. Marcos J. F. (2017). Mapping and identification of antifungal peptides in the putative antifungal protein AfpB from the filamentous fungus *Penicillium digitatum*. Front. Microbiol. 8:592. 10.3389/fmicb.2017.00592, PMID: 28428776 PMC5382200

[ref21] Ghag S. B. Shekhawat U. K. S. Ganapathi T. R. (2016). “Plant defensins for the development of fungal pathogen resistance in transgenic crops: production, safety, regulation and public health” in Genetically modified organisms in food. eds. Ronald W. Victor P. (Amsterdam, Netherlands: Elsevier Inc.), 381–396.

[ref22] Gow N. A. R. Latge J. -P. Munro C. A. De Groot P. W. J. Hellingwerf K. J. Klis F. M.. (2003). Cell wall architecture in yeast: new structure and new challenges minireview cell wall architecture in yeast: new structure and new challenges†. Yeast 9, 3341–3354. 10.1128/microbiolspec.FUNK-0035-2016

[ref23] Hayes B. M. E. Bleackley M. R. Wiltshire J. L. Anderson M. A. Traven A. van der Weerden N. L. (2013). Identification and mechanism of action of the plant defensin nad1 as a new member of the antifungal drug arsenal against *Candida albicans*. Antimicrob. Agents Chemother. 57, 3667–3675. 10.1128/AAC.00365-13, PMID: 23689717 PMC3719733

[ref24] Jampilek J. (2016). Potential of agricultural fungicides for antifungal drug discovery. Expert Opin. Drug Discovery 11, 1–9. 10.1517/17460441.2016.1110142, PMID: 26549424

[ref25] Kaewklom S. Euanorasetr J. Intra B. Panbangred W. Aunpad R. (2016). Antimicrobial activities of novel peptides derived from defensin genes of brassica hybrid Cv. pule. Int. J. Pept. Res. Ther. 22, 93–100. 10.1007/s10989-015-9488-2

[ref26] Laskowski R. A. MacArthur M. W. Moss D. S. Thornton J. M. (1993). PROCHECK: a program to check the stereochemical quality of protein structures. J. Appl. Crystallogr. 26, 283–291. 10.1107/S0021889892009944

[ref27] Laskowski R. A. Watson J. D. Thornton J. M. (2005). ProFunc: a server for predicting protein function from 3D structure. Nucleic Acids Res. 33, W89–W93. 10.1093/nar/gki414, PMID: 15980588 PMC1160175

[ref28] Latgé J. -P. (2007). The cell wall: a carbohydrate armour for the fungal cell. Mol. Microbiol. 66, 279–290. 10.1111/j.1365-2958.2007.05872.x, PMID: 17854405

[ref29] Lee J. Jung S. W. Cho A. E. (2016). Molecular insights into the adsorption mechanism of human β-defensin-3 on bacterial membranes. Langmuir 32, 1782–1790. 10.1021/acs.langmuir.5b04113, PMID: 26835546

[ref30] Letousey P. Marion D. de Zélicourt A. Thoiron S. Simier P. Elmorjani K.. (2007). Ha-DEF1, a sunflower defensin, induces cell death in orobanche parasitic plants. Planta 226, 591–600. 10.1007/s00425-007-0507-1, PMID: 17375322

[ref31] Li Y. L. Dai X. R. Yue X. Gao X. -Q. Zhang X. S. (2014). Identification of small secreted peptides (SSPs) in maize and expression analysis of partial SSP genes in reproductive tissues. Planta 240, 713–728. 10.1007/s00425-014-2123-1, PMID: 25048445

[ref32] Lin K. -F. Lee T. -R. Tsai P. -H. Hsu M. -P. Chen C. -S. Lyu P. -C. (2007). Structure-based protein engineering for alpha-amylase inhibitory activity of plant defensin. Proteins 68, 530–540. 10.1002/prot.21378, PMID: 17444520

[ref33] Malbrán I. Mourelos C. A. Girotti J. R. Aulicino M. B. Balatti P. A. Lori G. A. (2012). Aggressiveness variation of *Fusarium graminearum* isolates from Argentina following point inoculation of field grown wheat spikes. Crop Prot. 42, 234–243. 10.1016/j.cropro.2012.05.025

[ref34] Malbrán I. Mourelos C. A. Girotti J. R. Balatti P. A. Lori G. A. (2014). Toxigenic capacity and trichothecene production by *Fusarium graminearum* isolates from Argentina and their relationship with aggressiveness and fungal expansion in the wheat spike. Phytopathology 104, 357–364. 10.1094/PHYTO-06-13-0172-R, PMID: 24168045

[ref35] Malbrán I. Mourelos C. A. Girotti J. R. Lori G. A. (2018). “Trichothecenes” in Handbook of foodborne diseases. ed. Liu D. (Boca Raton: CRC Press), 977–986.

[ref36] Marchler-Bauer A. Lu S. Anderson J. B. Chitsaz F. Derbyshire M. K. DeWeese-Scott C.. (2011). CDD: a conserved domain database for the functional annotation of proteins. Nucleic Acids Res. 39, D225–D229. 10.1093/nar/gkq1189, PMID: 21109532 PMC3013737

[ref37] McCormick S. P. (2003). “The role of DON in pathogenicity” in Fusarium head blight of wheat and barley. eds. Leonard K. J. Bushnell W. R. (St. Paul, Minnesota: The American Phytopathological Society), 165–183.

[ref38] Muñoz A. Chu M. Marris P. I. Sagaram U. S. Kaur J. Shah D. M.. (2014). Specific domains of plant defensins differentially disrupt colony initiation, cell fusion and calcium homeostasis in neurospora crassa. Mol. Microbiol. 92, 1357–1374. 10.1111/mmi.12634, PMID: 24773060

[ref39] Nascimento V. V. D. Mello E. D. O. Carvalho L. P. De Melo E. J. T. Carvlho A. D. O. Fernandes K. V. S.. (2015). PvD1 defensin, a plant antimicrobial peptide with inhibitory activity against leishmania amazonensis. Biosci. Rep. 35:e00248. 10.1042/BSR20150060, PMID: 26285803 PMC4613715

[ref40] Niarchou A. Alexandridou A. Athanasiadis E. Spyrou G. (2013). C-PAmP: large scale analysis and database construction containing high scoring computationally predicted antimicrobial peptides for all the available plant species. PLoS One 8:e79728. 10.1371/journal.pone.0079728, PMID: 24244550 PMC3823563

[ref41] Ochiai A. Ogawa K. Fukuda M. Ohori M. Kanaoka T. Tanaka T.. (2018). Rice defensin OsAFP1 is a new drug candidate against human pathogenic fungi. Sci. Rep. 8:11434. 10.1038/s41598-018-29715-w, PMID: 30061724 PMC6065317

[ref42] Osborn R. W. De Samblanx G. W. Thevissen K. Goderis I. Torrekens S. Van Leuven F.. (1995). Isolation and characterisation of plant defensins from seeds of asteraceae, fabaceae, hippocastanaceae and saxifragaceae. FEBS Lett. 368, 257–262. 10.1016/0014-5793(95)00666-w, PMID: 7628617

[ref43] Parisi K. Shafee T. M. A. Quimbar P. van der Weerden N. L. Bleackley M. R. Anderson M. A. (2018). The evolution, function and mechanisms of action for plant defensins. Semin. Cell Dev. Biol. 88, 107–118. 10.1016/j.semcdb.2018.02.004, PMID: 29432955

[ref44] Perfect J. R. (2016). Is there an emerging need for new antifungals? Expert Opin. Emerg. Drugs 21, 129–131. 10.1517/14728214.2016.1155554, PMID: 26883732

[ref45] Powers J. -P. S. Tan A. Ramamoorthy A. Hancock R. E. W. (2005). Solution structure and interaction of the antimicrobial polyphemusins with lipid membranes. Biochemistry 44, 15504–15513. 10.1021/bi051302m, PMID: 16300399 PMC1386647

[ref46] Quarantin A. Hadeler B. Kröger C. Schäfer W. Favaron F. Sella L.. (2019). Different hydrophobins of *Fusarium graminearum* are involved in hyphal growth, attachment, water-air interface penetration and plant infection. Front. Microbiol. 10:751. 10.3389/fmicb.2019.00751, PMID: 31031728 PMC6474331

[ref47] Sagaram U. S. El-Mounadi K. Buchko G. W. Berg H. R. Kaur J. Pandurangi R. S.. (2013). Structural and functional studies of a phosphatidic acid-binding antifungal plant defensin MtDef4: identification of an RGFRRR motif governing fungal cell entry. PLoS One 8:e82485. 10.1371/journal.pone.0082485, PMID: 24324798 PMC3853197

[ref48] Sagaram U. S. Pandurangi R. Kaur J. Smith T. J. Shah D. M. (2011). Structure-activity determinants in antifungal plant defensins MsDef1 and MtDef4 with different modes of action against *Fusarium graminearum*. PLoS One 6:e18550. 10.1371/journal.pone.0018550, PMID: 21533249 PMC3076432

[ref49] Šali A. Blundell T. L. (1993). Comparative protein modelling by satisfaction of spacial restraints. J. Mol. Biol. 34, 779–815. 10.1006/jmbi.1993.1626, PMID: 8254673

[ref50] Sathoff A. E. Velivelli S. Shah D. M. Samac D. A. (2019). Plant defensin peptides have antifungal and antibacterial activity against human and plant pathogens. Phytopathology 109, 402–408. 10.1094/PHYTO-09-18-0331-R, PMID: 30252607

[ref51] Savary S. Willocquet L. Pethybridge S. J. Esker P. McRoberts N. Nelson A. (2019). The global burden of pathogens and pests on major food crops. Nat. Ecol. Evol. 3, 430–439. 10.1038/s41559-018-0793-y, PMID: 30718852

[ref52] Schaaper W. M. Posthuma G. A. Meloen R. H. Plasman H. H. Sijtsma L. Van Amerongen A.. (2001). Synthetic peptides derived from the Β2-Β3 loop of *Raphanus sativus* antifungal protein 2 that mimic the active site. J. Pept. Res. 57, 409–418. 10.1034/j.1399-3011.2001.00842.x, PMID: 11350601

[ref53] Schmitt P. Rosa R. D. Destoumieux-Garzón D. (2016). An intimate link between antimicrobial peptide sequence diversity and binding to essential components of bacterial membranes. Biochim. Biophys. Acta 1858, 958–970. 10.1016/j.bbamem.2015.10.011, PMID: 26498397

[ref54] Shafee T. M. A. Lay F. T. Hulett M. D. Anderson M. A. (2016). The defensins consist of two independent, convergent protein superfamilies. Mol. Biol. Evol. 33, 2345–2356. 10.1093/molbev/msw106, PMID: 27297472

[ref55] Silverstein K. A. T. Moskal W. A. Wu H. C. Underwood B. A. Graham M. A. Town C. D.. (2007). Small cysteine-rich peptides resembling antimicrobial peptides have been under-predicted in plants. Plant J. 51, 262–280. 10.1111/j.1365-313X.2007.03136.x, PMID: 17565583

[ref56] Slavokhotova A. A. Odintsova T. I. Rogozhin E. A. Musolyamov A. K. Andreev Y. A. Grishin E. V.. (2011). Isolation, molecular cloning and antimicrobial activity of novel defensins from common chickweed (*Stellaria media* L.) seeds. Biochimie 93, 450–456. 10.1016/j.biochi.2010.10.019, PMID: 21056078

[ref57] Söding J. Biegert A. Lupas A. N. (2005). The HHpred interactive server for protein homology detection and structure prediction. Nucleic Acids Res. 33, W244–W248. 10.1093/nar/gki408, PMID: 15980461 PMC1160169

[ref58] Spelbrink R. G. Dilmac N. Allen A. Smith T. J. Shah D. M. Hockerman G. H. (2004). Differential antifungal and calcium channel-blocking activity among structurally related plant defensins. Plant Physiol. 135, 2055–2067. 10.1104/pp.104.040873, PMID: 15299136 PMC520777

[ref59] Teixeira V. Feio M. J. Bastos M. (2012). Role of lipids in the interaction of antimicrobial peptides with membranes. Prog. Lipid Res. 51, 149–177. 10.1016/j.plipres.2011.12.005, PMID: 22245454

[ref60] Thevissen K. Kristensen H. -H. Thomma B. P. H. J. Cammue B. P. A. François I. E. J. A. (2007). Therapeutic potential of antifungal plant and insect defensins. Drug Discov. Today 12, 966–971. 10.1016/j.drudis.2007.07.016, PMID: 17993416

[ref61] Thomma B. P. H. J. Cammue B. P. A. Thevissen K. (2003). Mode of action of plant defensins suggests therapeutic potential. Curr. Drug Targets Infect. Disord. 3, 1–8. 10.2174/1568005033342000, PMID: 12570728

[ref62] van der Weerden N. L. Lay F. T. Anderson M. A. (2008). The plant defensin, NaD1, enters the cytoplasm of fusarium oxysporum hyphae. J. Biol. Chem. 283, 14445–14452. 10.1074/jbc.M709867200, PMID: 18339623

[ref63] Velivelli S. L. S. Islam K. T. Hobson E. Shah D. M. (2018). Modes of action of a bi-domain plant defensin MtDef5 against a bacterial pathogen xanthomonas campestris. Front. Microbiol. 9:934. 10.3389/fmicb.2018.00934, PMID: 29867843 PMC5964164

[ref64] Vijayan S. Singh N. K. Shukla P. Kirti P. B. (2013). Defensin (TvD1) from *Tephrosia villosa* exhibited strong anti-insect and anti-fungal activities in transgenic tobacco plants. J. Pest Sci. 86, 337–344. 10.1007/s10340-012-0467-5

[ref65] Vriens K. Cammue B. P. A. Thevissen K. (2014). Antifungal plant defensins: mechanisms of action and production. Molecules 19, 12280–12303. 10.3390/molecules190812280, PMID: 25153857 PMC6271847

[ref66] Vriens K. Cools T. L. Harvey P. J. Craik D. J. Spincemaille P. Cassiman D.. (2015). Synergistic activity of the plant defensin HsAFP1 and caspofungin against *Candida albicans* biofilms and planktonic cultures. PLoS One 10:e0132701. 10.1371/journal.pone.0132701, PMID: 26248029 PMC4527839

[ref67] Wegulo S. N. Baenziger P. S. Nopsa J. F. H. Bockus W. W. Hallen-Adams H. (2015). Management of fusarium head blight of wheat and barley. Crop Prot. 73, 100–107. 10.1016/j.cropro.2015.02.025

[ref68] Wiederstein M. Sippl M. J. (2007). ProSA-web: interactive web service for the recognition of errors in three-dimensional structures of proteins. Nucleic Acids Res. 35, W407–W410. 10.1093/nar/gkm290, PMID: 17517781 PMC1933241

[ref69] Yount N. Y. Yeaman M. R. (2004). Multidimensional signatures in antimicrobial peptides. Proc. Natl. Acad. Sci. U. S. A. 101, 7363–7368. 10.1073/pnas.0401567101, PMID: 15118082 PMC409924

