# Supplementary material for: Peptides Derived From the α-Core and γ-Core Regions of a Putative Silybum marianum Flower Defensin Show Antifungal Activity Against Fusarium graminearum
Source: Front Microbiol. 2021 Feb 17;12:632008. doi: 10.3389/fmicb.2021.632008 (PMC7925638; doi:10.3389/fmicb.2021.632008)
Supplement: Supplementary file 1 [file Data_Sheet_1.pdf]

## *Supplementary Material*

# Ramachandran Plot

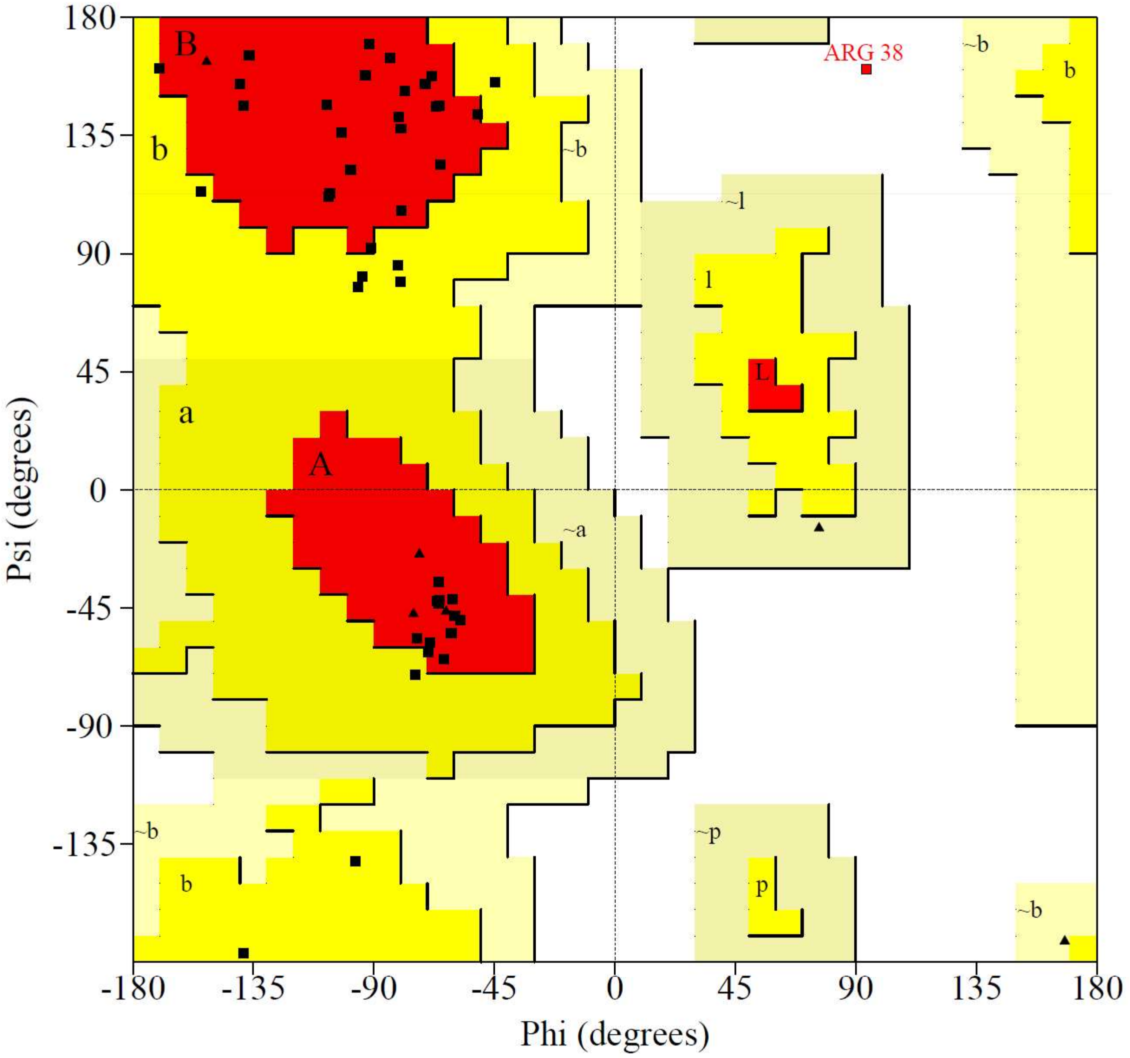


Plot statistics

| | | |
|---|---|---|
| Residues in most favoured regions [A,B,L] | 32 | 74.4% |
| Residues in additional allowed regions [a,b,l,p] | 10 | 23.3% |
| Residues in generously allowed regions [~a,~b,~l,~p] | 0 | 0.0% |
| Residues in disallowed regions | 1 | 2.3% |
| | ---- | ------ |
| Number of non-glycine and non-proline residues | 43 | 100.0% |
| Number of end-residues (excl. Gly and Pro) | 2 | |
| Number of glycine residues (shown as triangles) | 6 | |
| Number of proline residues | 3 | |
| | ---- | |
| Total number of residues | 54 | |

Based on an analysis of 118 structures of resolution of at least 2.0 Angstroms and R-factor no greater than 20%, a good quality model would be expected to have over 90% in the most favoured regions.

**Supplementary Figure 1.** Ramachandran plot and PROCHECK result for DefSm2-D homology model.

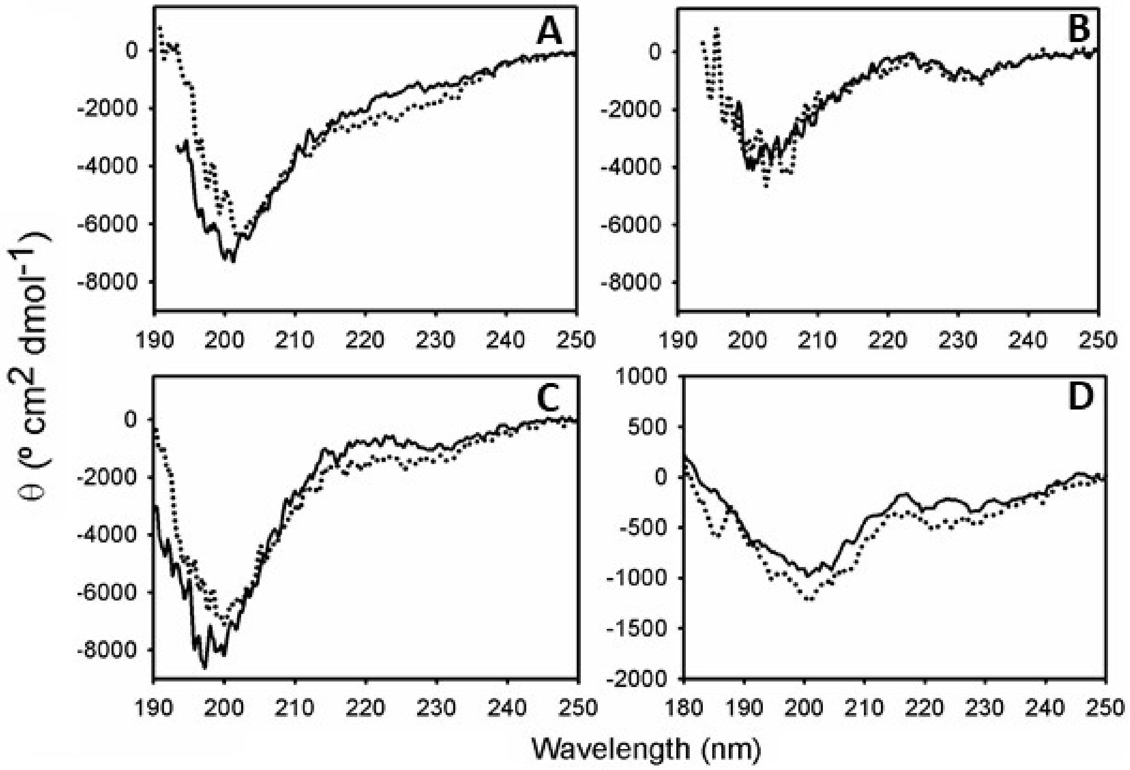


**Supplementary Figure 2.** Circular dichroism spectroscopy. Far UV CD spectra of each DefSm2-D derived peptide: SmAP$_{\alpha 1\text{-}21}$ (panel A), SmAP$_{\alpha 10\text{-}21}$ (panel B), SmAP$_{\gamma 27\text{-}44}$ (panel C) or SmAP$_{\gamma 29\text{-}35}$(panel D) are shown. Peptide samples were dissolved in water (solid line) or in 25% v/v aqueous TFE (dotted line).

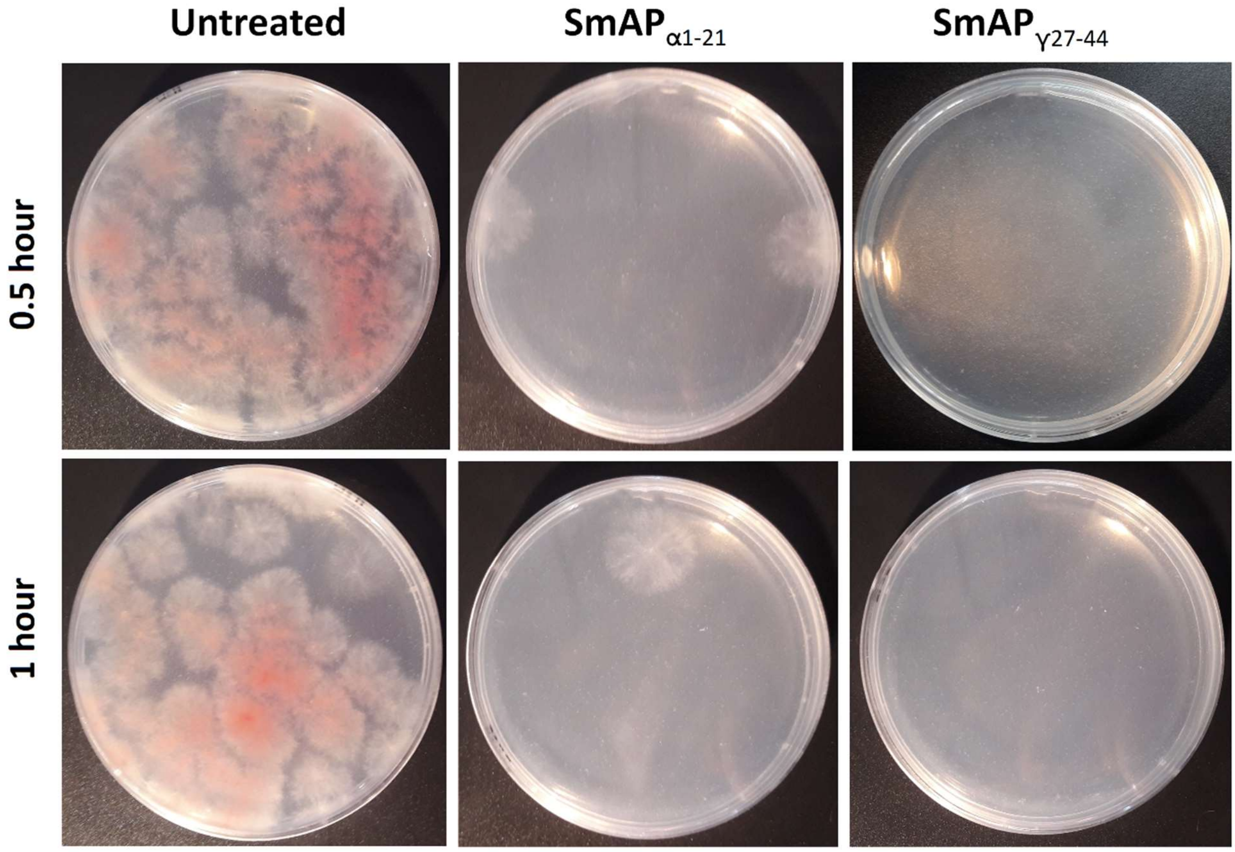


**Supplementary Figure 3.** *F. graminearum* growth in PD Agar plates incubated for 48 h at 25 °C in the presence of the peptides $SmAP_{\alpha 1\text{-}21}$ and $SmAP_{\gamma 27\text{-}44}$ as it was described for the time to kill experiment in Material and Methods. Two-time treatments are shown: 0.5 and 1 hour.
